# A mobile restriction modification system consisting of methylases on the IncA/C plasmid

**DOI:** 10.1186/s13100-019-0168-1

**Published:** 2019-06-07

**Authors:** Ruibai Wang, Jing Lou, Jie Li

**Affiliations:** State Key Laboratory for Infectious Disease Prevention and Control, National Institute for Communicable Disease Control and Prevention, Chinese Center for Disease Control and Prevention, Changbai Road 155, Changping, Beijing, 102206 People’s Republic of China

**Keywords:** Methylation, *Vibrio cholerae*, IncA/C plasmid, Cytosine-specific DNA methylase, Restriction modification system, Multidrug resistance

## Abstract

**Background:**

IncA/C plasmids play important roles in the development and dissemination of multidrug resistance in bacteria. These plasmids carry three methylase genes, two of which show cytosine specificity. The effects of such a plasmid on the host methylome were observed by single-molecule, real-time (SMRT) and bisulfite sequencing in this work.

**Results:**

The results showed that the numbers of methylation sites on the host chromosomes were changed, as were the sequences recognized by MTase. The host chromosomes were completely remodified by the plasmid with a methylation pattern different from that of the host itself. When the three *dcm* genes were deleted, the transferability of the plasmid into other *Vibrio cholerae* and *Escherichia coli* strains was lost. During deletion of the *dcm* genes, except for the wild-type strains and the targeted deletion strains, 18.7%~ 38.5% of the clones lost the IncA/C plasmid and changed from erythromycin-, azithromycin- and tetracycline-resistant strains to strains that were sensitive to these antibiotics.

**Conclusions:**

Methylation of the IncA/C plasmid was a new mobile restriction modification (RM) barrier against foreign DNA. By actively changing the host’s methylation pattern, the plasmid crossed the barrier of the host’s RM system, and this might be the simplest and most universal method by which plasmids acquire a broad host range. Elimination of plasmids by destruction of plasmid stability could be a new effective strategy to address bacterial multidrug resistance.

**Electronic supplementary material:**

The online version of this article (10.1186/s13100-019-0168-1) contains supplementary material, which is available to authorized users.

## Background

Antibiotic resistance, especially multidrug resistance, is a serious challenge worldwide. Many bacterial drug resistance genes are stored in plasmids and can be horizontally transferred to other bacteria. The IncA/C family plasmids are major members of these plasmids [1, 2]. Both of the famous plasmids, namely, pIP1202, which exhibits high resistance to at least eight antibiotics and was isolated from *Yersinia pestis* in 1995 [3], and the NDM-1 plasmid, which was isolated from superresistant bacteria in India, Bangladesh, Pakistan, Britain and the United States in August 2010 [4], are IncA/C plasmids. These plasmids have generated much concern with regard to public health and bioterrorism. IncA/C plasmids have the ability to robustly accumulate antibiotic resistance genes. There are many resistance genes against rifampicin, erythromycin, streptomycin, chloramphenicol, sulfonamides and disinfectants that are routinely harbored on these plasmids [5], in addition to a variety of beta-lactamase genes harbored by the pNDM-1_Dok01, pNDM102337, pNDM10469, pNDM10505, pNDMCFuy, pNDM-KN and pNDM-US plasmids of *Escherichia coli* and *Klebsiella pneumoniae*. The pVC211 plasmid that we isolated from *Vibrio cholerae* has 16 antibiotic resistance-related genes, including five macrolide resistance genes that utilize three mechanisms [6]. The IncA/C plasmid made 99% of Chinese *V. cholerae* O139 strains resistant to more than three antibiotics, and 47% resistant to eight antibiotics [7].

Another striking feature of the IncA/C plasmids is their wide host range. These plasmids exist and are transferred horizontally in many bacterial species and genera, such as *E. coli*, *Salmonella, Enterobacter*, *Klebsiella, V. cholerae*, *Yersinia*, *Pantoea, Edwardsiella*, *Citrobacter freundii*, *Photobacterium damselae, Aeromonas*, *Xenorhabdus nematophila,* and *Providencia,* with transformation efficiencies as high as 10^− 1^ to 10^− 2^. In addition, the skeleton of the IncA/C plasmid is also widely distributed in agricultural multidrug-resistant pathogens. Strains carrying IncA/C plasmids have been isolated from cows, chickens, turkeys and pigs. Reports have shown that the *bla*CMY-2 gene on the IncA/C plasmid that confers resistance to cephalosporin could be identified in human *E. coli* and *Klebsiella pneumoniae* isolates several years after the prevalence of the gene in edible animals [8]. Moreover, 1% of *E. coli* strains isolated from healthy people who had never taken antibiotics were positive for the *repA*/*C* gene, the replicator of the IncA/C plasmid [9, 10]. This mobile reservoir of drug resistance genes can transmit multidrug resistance phenotypes from foodborne pathogens to human pathogens, which demonstrates that the use of veterinary drugs can affect human drug resistance profiles, and the IncA/C plasmids have special public health implications.

IncA/C plasmids are large, conjugative plasmids that are approximately 150 kb in length. On the plasmid backbones, there are three methylation-related genes: *dcm1*, *dcm2*, and *dcm3*, which are 1626 bp, 1428 bp and 924 bp in length and are identical to the NCBI reference sequences WP_000201432.1**,** WP_000936896.1 and WP_000501488.1, respectively. The *dcm1* gene encodes Gammaproteobacteria cytosine-C5 specific DNA methylase (MTase) of the Pfam PF00145 family. The *dcm2* gene is a DNA cytosine methyltransferase with an AdoMet_MTase (cl17173) conserved domain. The *dcm3* gene is a DNA modification MTase that lacks any known conserved domain. These three methylation genes are broadly conserved in IncA/C_2_ plasmids, although a few plasmids have lost the *dcm2* gene due to a deletion event associated with the ARI-B resistance island [2]. There are six methylation-related genes in the host genome of *V. cholerae*: three adenine-specific MTases (*dam*: VC1769, VC2118, and VC2626), two rRNA MTases (VC2697 and VCA0627), and one orphan cytosine MTase, *vchM* (VCA0158). Generally, DNA (cytosine-5-specific) MTase (Dcm) recognizes the CCWGG motif and covalently adds a methyl group at the C5 position of the second cytosine, while Dam introduces a methyl group at the N6 position of the adenine in the GATC motif. The MTases encoded in the *V. cholerae* chromosomes are mainly Dam MTases, and the only Dcm MTase, VchM, recognizes a novel target, the first 5’C on both strands of the palindromic sequence 5′-RCCGGY-3′, and leaves all 5′-CCWGG-3′ sites unmethylated in *V. cholerae* [11]. These data suggest that the changes in DNA methylation mediated by the IncA/C plasmids may be different from those in the host chromosomes.

DNA methylation is a central epigenetic modification in various cellular processes, including DNA replication and repair [12], transcriptional modulation, lowering of transformation frequency, and stabilization of short direct repeats in certain bacteria; in addition, DNA methylation is necessary for site-directed mutagenesis [13]. DNA methylation acts either alone or as a part of the bacterial restriction modification (RM) system that protects the host from infection of foreign DNA and bacteriophages by degrading nonmethylated DNA with sequence-specific restriction enzymes. Although the role of IncA/C plasmids in drug resistance is well elucidated, the role of the *dcm* genes on A/C_2_ plasmids remains largely unexplored. In this study, the IncA/C plasmid pVC211 (148,456 bp) [6] was conjugated into *V. cholerae* strain C6706. Genome-wide bisulfite sequencing and single-molecule, real-time (SMRT) sequencing [14, 15] were conducted to provide a global view of the changes in the methylation patterns of the host DNA that were induced by this plasmid.

## Results

### Methylomes determined by SMRT sequencing

The two samples, C6706 and CV2, produced 998,990,159 bp and 1,097,369,564 bp polymerase reads, respectively, in SMRT sequencing, and the average sequencing coverage reached ~250x. In the C6706 genome, 1,476,420 methylation sites were detected, and 1490,035 methylation sites were detected in CV2. Although the actual sequencing coverages were much higher than the coverage required for ^m5^C analysis by PacBio RS II, the confidence of detection was not high enough, leading to failure of ^m5^C detection. In addition to the ^m4^C and ^m6^A sites, a large proportion of methylation sites, including ^m5^C, in both C6706 and CV2 were reported as ‘modified bases’ and failed to show definitive methylation patterns in SMRT analysis. The number of ^m4^C and ^m6^A methylation sites, whether intergenic or in coding sequences (CDSs), decreased after the transformation of the pVC211 plasmid, but the number of untyped methylation sites increased by 18,763 sites, resulting in a slight increase in the total methylation rate from 36.6% in C6706 to 36.94% in CV2 (Table 1).

**Table 1:** Methylation information derived from SMRT sequencing

	Total methylation sites		^m4^C			^m6^A			Untyped		
	C6706	CV2	C6706	CV2	Difference	C6706	CV2	Difference	C6706	CV2	Difference
CDS	192,116	187,228	151,109	146,542	− 4567	41,007	40,686	−321			
Intergenic	5594	5407	4362	4209	− 153	1232	1198	−34			
Total	1,475,558	1,490,035	155,471	151,472	− 3999	42,239	41,952	− 287	1,277,848	1,296,611	+ 18,763
Modification ratio (%)	36.58	36.94	3.85	3.76	−0.09	1.05	1.04	−0.01	31.68	32.14	0.46

There were 4013 annotated genes on the *V. cholerae* chromosome, including 2775 functional genes, 25 rRNA genes, and 94 tRNA genes distributed on chromosome I and 1115 functional genes and 4 tRNA genes distributed on chromosome II (Table 2). The methylation patterns on the two chromosomes of *V. cholerae* were considerably different. The genes on chromosome I were generally methylated, dominated by ^m4^C-^m6^A double methylation, and sites involving ^m4^C and ^m6^A accounted for 84.7% (3401/4013) of the total genes. On the other hand, more than half of the genes on chromosome II exhibited non-^m4^C non-^m6^A methylation. In particular, the methylation of the tRNA gene was the most significantly different between the two chromosomes, with 92 of the 94 genes on chromosome I involved in the methylation of ^m4^C or ^m6^A, while on chromosome II, only one of the four genes, VC_At2, was ^m4^C methylated. Chromosome II of *V. cholerae* is a mega-plasmid ameliorated to the host chromosome as a result of its long-standing presence in the host lineage [16]. The differences in the methylation patterns of chromosome II also demonstrate its heterogeneity with chromosome I. However, there was no obvious difference between C6706 and CV2 in terms of the methylation patterns of the two chromosomes.

**Table 2:** Methylation patterns of the two chromosomes of C6706 and CV2

Chromosome	Gene code	Gene number	Single ^m4^C		Single ^m6^A		^m4^C and ^m6^A		none ^m4^C/^m6^A	
			C6706	CV2	C6706	CV2	C6706	CV2	C6706	CV2
I	VC	2775	157	165	2	6	2602	2588	14	16
	VC_r	25	8	6	0	0	17	19	0	0
	VC_t	94	48	52	0	1	42	39	2	2
II	VC_A	1115	177	186	8	8	339	331	591	590
	VC_At	4	1	1	0	0	0	0	3	3
	Total	4013	391	410	10	15	3000	2977	610	611

However, the methylation sites on the genomes of C6706 and CV2 showed significant changes after conjugation of the IncA/C plasmid (Table 3). A total of 124,571 sites, consisting of 85,972 ^m4^C and 38,599 ^m6^A sites, in 3341 genes kept the same methylation type between the two samples. C6706 had 67,545 methylation sites that differed from CV2, and CV2 had 62,657 specific methylation sites. These differential sites accounted for 37.46 and 35.16% of the C6706 and CV2 methylation sites, respectively, involving approximately 81% of the total genes (97% of the total genes on chromosome I and 40% on chromosome II). This large-scale methylation change made it impossible to discern any gene function modification preference. Among the genes with differential methylation sites, the methylation modes of 116 genes changed significantly (Additional file 1: Table S2); for example, the methylation pattern of VC_0025 changed from ^m4^C-^m6^A double methylation to ^m4^C single methylation, that of VC_A0698 changed from ^m4^C methylation to ^m6^A methylation, and that of VC_1030 changed from ^m4^C methylation to non-^m4^C non-^m6^A methylation. Although 64 genes encoded hypothetical proteins with unknown function, other differentially methylated genes involved many key processes in the lifecycle of the *V. cholerae* host. These genes included the following: 1 LuxR family transcriptional regulator; 3 LysR family transcriptional regulators; 5 regulatory proteins related to phosphoglycerate transport, cold shock, sigma-54 dependent transcription, response and mannitol metabolism; Hcp-1, which is involved in chromosome segregation; 7 transport and binding proteins related to Na+/H+, vibriobactin and enterobactin transportation; and 5 proteins related to protein synthesis and cell fate.

**Table 3:** Common and differential ^m4^C and ^m6^A methylation sites in CDS between C6706 and CV2

Chromosome	Gene code	Gene number	Common methylation sites			Differential methylation sites					
			m4C	m6A	Total number (involved genes)	m4C		m6A		Total number (involved genes)	
						C6706	CV2	C6706	CV2	C6706	CV2
I	VC	2775	80,978	36,609	117,587 (2744)	61,330	56,919	2203	1882	63,533 (2715)	58,801 (2700)
	VC_r	25	1697	419	2116 (25)	1093	1082	56	94	1149 [23]	1176 (24)
	VC_t	94	314	70	384 (85)	236	231	12	6	248 (79)	237 (82)
II	VC_A	1115	2980	1501	4481 (486)	2476	2335	137	105	2613 (464)	2440 (447)
	VC_At	4	3	0	3 (1)	2	3	0	0	2 (1)	3 (1)
Total		4013	85,972	38,599	124,571 (3341)	65,137	60,570	2408	2087	67,545 (3282)	62,657 (3254)

Analysis of the motif sequences using the P_MotifFind Module of SMRT further revealed the effects of the plasmid-encoded MTases on the methylation status of the host chromosomes (Table 4). Eleven and ten motifs were detected in C6706 and CV2, respectively, but only 6 motifs were commonly detected in both samples, and these shared motifs also showed significant differences in number and proportion between the two samples. Four motifs found in C6706, namely, AGKNNNNW, GADNDGCG, GARVNNDG, and TVVVNNDG, were changed to ABNBMVBW, GANNDBBG, GARVNRNG and TNVVNNDG in CV2, respectively. The DCAGVHRNG motif that was present in C6706 disappeared in CV2. These data indicate that plasmid transformation not only changed the methylation types of the host chromosomes, but also changed the characteristics of the identification sequences of the MTases.

**Table 4:** Motifs in C6706 and CV2 as determined by SMRT sequencing

Types	Motif	nGenome^a^		^m6^A		^m4^C		Modified_base		Undetected modification sites	
		C6707	CV2	C6707	CV2	C6707	CV2	C6707	CV2	C6707	CV2
Common (6 types)	AAGNNNNNNCATC	454	532	452 (99.6%)	528 (99.2%)	0	0	1 (0.2%)	0	1 (0.2%)	4 (0.8%)
	AKDTRGCA	386	476	181 (46.9%)	244 (51.3%)	0	0	168 (43.5%)	186 (39.1%)	37 (9.6%)	46 (9.7%)
	AKGYANYA	755	456	300 (39.7%)	171 (37.5%)	0	0	260 (34.4%)	93 (20.4%)	195 (25.8%)	192 (42.1%)
	GATC	37,401	34,375	37,070 (99.1%)	33,991 (98.9%)	0	0	71 (0.2%)	95 (0.3%)	260 (0.7%)	289 (0.8%)
	GATGNNNNNNCTT	613	562	609 (99.3%)	558 (99.3%)	0	0	4 (0.7%)	3 (0.5%)	0	1 (0.2%)
	TNNNNNNH	348,498	334,917	0	0	0	0	220,207 (63.2%)	213,464 (63.7%)	128,291 (36.8%)	121,453 (36.3%)
C6707 specific (5 types)	AGKNNNNW	5445	0	2795 (51.33%)	0	0	0	1727 (31.7%)	0	923 (17.0%)	0
	GADNDGCG	2993	0	0	0	0	0	1403 (46.9%)	0	1590 (53.1%)	0
	GARVNNDG	13,031	0	0	0	0	0	8830 (67.8%)	0	4201 (32.2%)	0
	TVVVNNDG	26,727	0	0	0	0	0	15,684 (58.7%)	0	11,043 (41.3%)	0
	DCAGVHRNG	1490	0	0	0	476 (31.9%)	0	665 (44.6%)	0	349 (23.4%)	0
CV2 specific (4 types)	ABNBMVBW	0	10,259	0	6318 (61.8%)	0	0	0	2596 (25.3%)	0	1345 (13.1%)
	GANNDBBG	0	14,158	0	0	0	0	0	8130 (57.4%)	0	6028 (42.6%)
	GARVNRNG	0	7699	0	0	0	0	0	4448 (57.8%)	0	3251 (42.2%)
	TNVVNNDG	0	27,441	0	0	0	0	0	16,388 (59.7%)	0	11,053 (40.3%)

^a^ Number of this motif in the reference sequence genome

### ^m5^C methylation in bisulfite sequencing

**Fig. 1:**
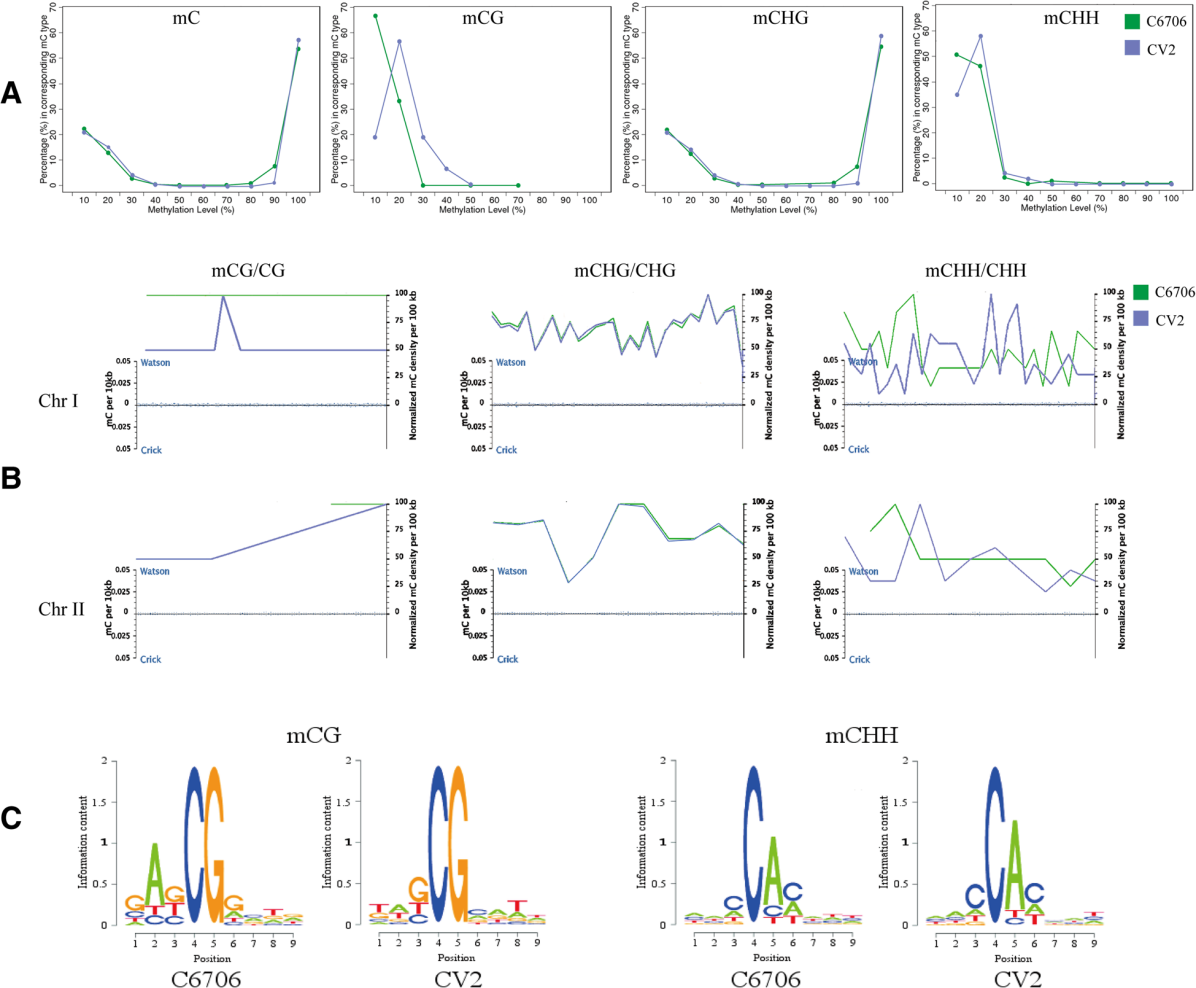
Global trends of the methylomes of C6706 and CV2 and logo plots of the non-CG methylation patterns. A. Distribution of the methylation level in the C6706 and CV2 samples. The x-axis indicates the methylation level, and the y-axis indicates the fraction of mC at a specific methylation level in all methylcytosines. The methylation level of cytosine is the proportional value of the sequence supporting the C base site as a methylation site in the effective coverage sequence. B. The density distribution of mC on each chromosome. The x-axis represents the chromosome. The y-axis on the left represents the mC density calculated from a 10 kb window, and the blue dot represents the distribution of the mC density on the chromosome. The y-axis on the right represents the normalized mC ratio. The curves represent the density distribution of the different types of mC bases (CG, CHG and CHH). C. Logo plots of the sequence features of the adjacent bases at the C site. The x-axis represents the base position, with the C-base being analyzed in the fourth position. The y-axis represents the entropy value

Because of the failure of ^m5^C site detection in SMRT, we had to use the traditional ^m5^C analysis method, bisulfite sequencing. Samples C6706 and CV2 produced 16,196,082 bp and 16,232,236 bp clean reads, respectively. The mapping rates were 96.01 and 91.78%; the bisulfite conversion rates were 99.65 and 99.62%; and the average depths were 362.12x and 345.69x, respectively. We detected 6965 methylcytosines in C6706 and 7321 methylcytosines in CV2 from a total of 1,915,376 cytosines in the *V. cholerae* genome. This increase in the number of methylcytosines in CV2 was consistent with the increase in untyped methylation sites in SMRT sequencing and the cytosine-specificity of the MTases on the IncA/C_2_ plasmid. The average methylation levels of the three types of C bases (mCG, mCHG and mCHH, where H = A, C or T) were higher in CV2 than in C6706. At the whole-genome level, the mCG, mCHG and mCHH methylation levels were 0.33, 1.74 and 0.43% in C6706, respectively, and 0.34, 1.81 and 0.44% in CV2, respectively. Moreover, the graphs of the methylation levels also showed a slight increasing trend from C6706 to CV2 (Fig. 1A). For example, 50% of the mCHH sites were 10% methylated in C6706; it were 59% of the mCHH sites 20% methylated in CV2. Notably, although the total number of mCG sites was the most similar between C6706 and CV2, the methylation levels of these sites were greatly different. The methylation levels of the mCG sites changed as follows: 68% of the mCG sites were 10% methylated and 32% of the mCG sites were 20% methylated in C6706; 19% of the mCG sites were 10% methylated, 57% of the mCG sites were 20% methylated, and 18% of the mCG sites were 30% methylated in CV2 (Fig. 1a).

The global-scale view of the DNA methylation density in 100-kb windows (Fig. 1b) revealed that there was no correlation between the distributions of the three methylation types on the two chromosomes of *V. cholerae*. The density of mCG methylation was relatively steady in each chromosomal region, while the non-CG methylation levels fluctuated greatly throughout each chromosome. In C6706 and CV2, the smooth profiles of the mCHG density remained constant, but the mCHH density profiles changed dramatically without any similarity (Fig. 1b).

The sequence characteristics of the bases in the vicinity of a methylation site reflect the preference of MTases for recognition of specific sequences, so we calculated the methylation percentage of the nine bases upstream and downstream of the methylation site (mC at the fourth base). The enrichment of particular local sequences was observed for both mCG and non-CG methylcytosine (Additional file 2: Figure S1). 5′-RCCGGY-3′ was the recognition sequence of mCHG, and preferences for an AG dinucleotide of mCG and a C of mCHH upstream of the methylation sites were observed. This result was consistent with the analysis of *vchM* [11], but also showed that in addition to the 5′-RCCGGY-3′ sequence, although the 5′-CCWGG-3′ sites were unmethylated, other ^m5^C methylated sequences existed in *V. cholerae*. C6706 and CV2 were identical in this sequence preference, but there were differences in the relative frequency of the bases before and after the recognition sites of mCG and mCHH (Fig. 1c).

In C6706, 6833 ^m5^C methylation sites were located in 1436 genes on chromosome I and in 446 genes on chromosome II, accounting for 49.60 and 39.85% of the total genes of the two chromosomes, respectively, and included 459 ^m5^C sites in intergenic regions. In CV2, 6851 ^m5^C methylation sites were located in 1554 genes on chromosome I, 492 genes on chromosome II, and 471 sites in intergenic regions. Between the two samples, 6466 ^m5^C methylation sites remained constant. There were 499 differential ^m5^C sites in C6706 and 856 sites in CV2, involving 377 and 635 genes, respectively. There were almost no identical mCHH methylation sites between C6706 and CV2, similar to the mCG sites on chromosome II of the two strains (Table 5). Overall, DNA methylation in non-CG contexts (mCHG and mCHH) accounted for an absolute majority (99.87 and 99.78%) in *V. cholerae* and accounted for most of the differences in methylation before and after plasmid transformation.

**Table 5:** Three methylation types of C bases in bisulfite sequencing between C6706 and CV2

Chromosome	Location	Constant			Differential					
		mCG	mCHG	mCHH	mCG		mCHG		mCHH	
					C6706	CV2	C6706	CV2	C6706	CV2
I	gene	3	4674	1	2	4	269	458	54	110
	intergenic	1	263	0	2	3	37	30	15	18
II	gene	0	1400	0	4	3	76	159	23	39
	intergenic	0	124	0	1	2	10	19	6	11
	total	4	6461	1	9	12	392	666	98	178

### Methylation of host MTase genes

Four of the six MTase genes on the *V. cholerae* chromosomes had ^m4^C and ^m6^A methylated sites; VC1769 was the only gene that had all three methylation types. We did not detect any typed methylation sites on VCA0627 or on the orphan Dcm gene, VCA0158 (Table 6). After transformation with pVC211, only a few ^m6^A sites changed, while more than half of the ^m4^C sites changed. Therefore, it was reasonable to observe the large-scale change in the methylation profile of the host chromosomes by SMRT sequencing. Combined with changes in ^m5^C sites determined by bisulfite sequencing, our results suggested that changes in the host methylation profile were partly attributable to the indirect role of host MTases that were affected by the plasmid and were partly attributable to the direct role of plasmid-encoded MTases themselves.

**Table 6:** Methylation of the six host MTase genes

MTase	Gene	^m4^C			^m6^A			^m5^C		
		Constant	Differential		Constant	Differential		Constant	Differential	
			C	CV2		C	CV2		C	CV2
Dam	VC1769	39	29	44	22	3	6	2	0	0
	VC2118	19	25	19	16	0	1	–	–	–
	VC2626	16	40	22	9	0	0	–	–	–
rRNA MTase	VC2697	18	42	19	17	0	1	–	–	–
	VCA0627	–	–	–	–	–	–	–	–	–
Dcm (vchM)	VCA0158	–	–	–	–	–	–	–	–	–

### Knockouts of the three MTase genes on pVC211

All seven types of VC211 mutants, including single, two and three *dcm* gene deletions, were constructed successfully, which indicated that none of the three methylase genes were essential for pVC211. However, among the secondary homologous recombinants selected from the sucrose plates to test for the gene deletion, several clones lost pVC211, except for the wild-type strains and the target gene-deleted strains. These clones were positive for amplification with the *ctxAB*-U/L primer set and negative for the del *dcm*1/4 and *repA*-F/R primer sets. To confirm this phenomenon, all seven types of gene knockouts were repeated twice. The highest plasmid loss rate was found in the *dcm1* single-gene deletion, with an average of 37/96 (the wild-type rate was 14/96, and the gene deletion rate was 45/96). Among the other five types of deletions, the plasmid loss rate was approximately 18/96, while plasmid loss was not observed in the *dcm2* single-gene deletion. Accompanied with the loss of this IncA/C plasmid, the *V. cholerae* strain VC211 changed from a strain with erythromycin (MIC> 128 μg/ml), azithromycin (MIC≥64 μg/ml) and tetracycline (16 μg/ml) resistance [6] to a sensitive strain.

The loss of pVC211 in the deletion process of the *dcm* genes indicated that the *dcm* genes might affect the plasmid stability, so the presence of plasmids was detected by PCR in the 96 clones of the VC211∆*dcm1* and VC211∆(*dcm1*,*dcm2*,*dcm3*) mutant strains after three continuous passage cultures in 5 ml Luria–Bertani (LB) broth without any antibiotics; PCR was performed with the *repA*-F/R primer set as described previously [7]. None of the progeny clones that lost the plasmid were detected, and the results show that the IncA/C plasmids with the three *dcm* genes deleted continued to exist stably in host *V. cholerae* strains.

The transferability of pVC211∆(*dcm1*,*dcm2*,*dcm3*) between the *V. cholerae* strains was tested. VC 211∆(*dcm1*,*dcm2*,*dcm3*) was conjugated with N16961 (*lacZ*::kan^r^), C6706 (*lacZ*::kan^r^), VC2981 and VC2973 and then spread onto LB plates containing two antibiotics using VC211 as a control. The results show that the pVC211 plasmid lacking the three *dcm* genes could no longer be transferred to other *V. cholerae* strains. Furthermore, freshly cultured SM10 strains were divided into two aliquots and conjugated with VC211∆(*dcm1*,*dcm2*,*dcm3*) and VC211 to test the plasmid transferability between the *V. cholerae* and *E. coli* strains. The results were interesting. The clones grown on the double antibiotics LB plates of the SM10 and VC211 conjugation were positive for *repA*-F/R amplification and negative for *ctxAB*-U/L amplification, indicating that the pVC211 plasmid had been transferred into SM10. All of the clones grown on the plates of the conjugation of SM10 and VC211∆(*dcm1*,*dcm2*,*dcm3*) were positive for *repA*-F/R and *ctxAB*-U/L amplification. These clones were also positive for the kan-mini-U/L amplification of mini-Tn5 and were resistant to kanamycin (100 μg/mL), indicating that instead of pVC211∆(*dcm1*,*dcm2*,*dcm3*) being transferred into SM10, the mini-Tn5 transposon of SM10 [17] was transferred into *V. cholerae*.

## Discussion

Methylation plays important roles in epigenetic gene regulation in both eukaryotic and prokaryotic organisms. Compared with the explicit function of Dam methylation in mismatch repair, initiation of chromosome replication, transcription regulation at promoters containing GATC sequences, gene expression, pathogenicity and DNA stability under antibiotic pressure [18], knowledge of the function of Dcm MTase is limited [2]. The original function elucidated for Dcm was to discriminate self DNA from foreign DNA as part of the RM system [19] and to affect the plasmid transfer efficiency and dissemination of antibiotic resistance genes in *Enterococcus faecalis* [15]. Therefore, the discovery that two of the three MTase genes on the IncA/C_2_ plasmid are cytosine-specific was intriguing.

Three types of methylation, namely, ^m6^A, ^m4^C and ^m5^C, and a large amount of methylation without obvious methylation patterns exist in *V. cholerae*. Comparison of the genome-wide methylation profiles of *V. cholerae* C6706 before and after the conjugation of pVC211 revealed that the MTases of the IncA/C plasmid changed the number and characteristics of all types of methylation. The changes involved most of the regions of the two chromosomes, and no obvious preference for gene function was observed. It seemed that the host chromosomes were completely relabeled by the plasmid with a methylation pattern different from that of the host itself. Surprisingly, the new methylation pattern induced by the plasmid could be tolerated by the restriction enzymes of the host’s RM system.

After loss of the three *dcm* genes, pVC211 could not be transferred into other *V. cholerae* and *E. coli* strains as the wild-type plasmid, which demonstrated that instead of mimicking the host’s methylation pattern [20], pVC211 crossed the barrier of the host’s RM system by actively changing the host’s methylation pattern. This is the simplest and most general method by which IncA/C plasmids obtain their broad host range. Although pVC211∆(*dcm1*,*dcm2*,*dcm3*) could no longer be transferred, this plasmid could still stably exist in the host strain when there was no selection pressure. We speculated that once the plasmid entered the host, other genes on the plasmid participated in plasmid maintenance, and methylation was no longer the only determinant of plasmid stability. After deletion of the *dcm* genes, the mini-Tn5 transposon of SM10 could enter VC211. Combined with the methylation of the host chromosomes by the plasmid, this finding suggested that the MTases of the plasmid actually constructed a new RM barrier to nonself DNA for itself. In addition to the bacterial RM for foreign DNA on chromosomes, the mobile genetic elements could also carry their own barriers. Moreover, transfer of the suicide plasmid pWM91 into VC211 clearly demonstrated the selectivity of the IncA/C RM barrier.

In addition, it is worth noting the loss of the IncA/C plasmid during the deletion of the *dcm* genes. In theory, after the first recombination, the suicide plasmid pWM91 was inserted into pVC211, and it was possible that the pVC211 plasmid was expelled with pWM91 due to the toxic effect of the *sacB* gene on the sucrose plate. However, IncA/C plasmids were very stable in *V. cholerae* [7], and in the previous deletions of plasmid genes [6], only the secondary recombined wild-type strains and the gene deletion strains were obtained after sucrose selection. The loss of the IncA/C plasmid together with the suicide plasmid never occurred as it did in the deletion of the *dcm2* gene in this study. Therefore, even the *dcm* genes were not the determinants of plasmid stability, and after the deletion of the *dcm* genes, the plasmids continued to exist stably in the subcultures, the loss of plasmid caused by the *dcm* gene deletion indicated that these genes could possibly contribute to the stability of the plasmids. Loss of the IncA/C plasmids transformed the host bacteria from plasmid-conferred antibiotic resistant strains to sensitive strains. This result indicated that elimination of plasmids by destruction of plasmid stability could be a new, effective strategy to address bacterial multidrug resistance.

## Conclusions

MTases of the IncA/C plasmid were a mobile RM system of plasmids against nonself DNA, including host chromosomes and other mobile genetic elements. By actively changing the host’s methylation pattern, this system helped the plasmids cross the barrier of the host’s RM system and enabled the broad host range of the plasmids.

## Materials and methods

### Strains and plasmids

The toxigenic O1 El Tor strain C6706 and N16961 are commonly used *V. cholerae* reference strains, isolated in 1991 from Peru [21] and 1971 from Bangladesh, respectively [22]. VC2981 and VC2973 are nontoxigenic O1 El Tor strains with ampicillin resistance isolated from Jiangsu Province in 1965. The IncA/C_2_ plasmid pVC211 was identified in a toxigenic O139 serogroup strain VC211 isolated in 2003 from Guangdong Province, China [6].

### Construction of the kanamycin-resistant mutants of C6706 and N16961

The kanamycin resistance gene was amplified from the plasmid pet-ken with the kan-844-stuI-U/L primer set and was digested with the enzyme *stu*I; then, this gene was ligated to the vector pJL-1, which was also digested with *stu*I, and transformed into the *Escherichia coli* strain SM10. By conjugation and via the homologous arms of the *lacZ* genes on pJL-1, a kanamycin (kan) resistance gene was inserted into the genomes of the *V. cholerae* strains N16961 (streptomycin, str^r^) and C6706 (str^r^). Then, N16961 (*lacZ*::kan^r^, str^r^) and C6706 (*lacZ*::kan^r^, str^r^) were constructed. Furthermore, the pVC211 plasmid was transferred into C6706 (*lacZ*::kan^r^, str^r^) by conjugation, and the strain CV2 was constructed.

### Bisulfite sequencing

Genomic DNA was fragmented into ~ 250-bp fragments using Bioruptor (Diagenode, Belgium). The fragments were blunt ended, phosphorylated, subjected to 3′-dA overhang generation and ligated to methylated sequencing adaptors. Then, the samples were treated with bisulfite by using the EZ DNA Methylation-Gold Kit (Zymo Research, Inc., Irvine, CA, USA), desalted and size selected by 2% agarose gel electrophoresis. After PCR amplification and another round of size selection, the qualified libraries were sequenced. The sequencing data were filtered, and the low-quality data were removed. Then, the clean data were mapped onto the reference genome (the complete genome sequences of the standard strain N16961, AE003852 and AE003853; BSMAP) to obtain the methylation information for all cytosines throughout the genome for standard and personalized bioinformatic analysis. The average methylation level was calculated as follows:

$$ {\mathrm{Rm}}_{\mathrm{average}}=\frac{{\mathrm{Nm}}_{\mathrm{all}}}{{\mathrm{Nm}}_{\mathrm{all}}+{\mathrm{Nnm}}_{\mathrm{all}}}\times 100\% $$

where Nm is the number of methylated cytosines, and Nnm is the number of reads with nonmethylated cytosines. Windows containing at least 5 CG (CHG or CHH) at the same location in the two genomes were identified, and the difference in the level of CG methylation in these windows between the two samples was compared to find regions with significant differences in methylation (differentially methylated region, DMR; 2-fold difference, Fisher test *P* value ≤0.05). If two adjacent DMRs formed a contiguous region with significantly different methylation levels in both samples, the two DMRs were merged into one continuous DMR; otherwise, these DMRs were considered to be separate.

CIRCOS was used to compare the differences in the methylation levels of DMRs between the samples. The degree of difference in the methylation level at one site in the two samples was calculated by the following formula, where Rm1 and Rm2 represent the mC methylation level of the two samples. If the value of either Rm1 or Rm2 was 0, it was replaced with 0.001 [23].

$$ \mathrm{Degree}\ \mathrm{of}\ \mathrm{difference}=\frac{\log_2\mathrm{Rm}1}{\log_2\mathrm{Rm}2} $$

### SMRT sequencing

First, the target fragments were amplified from the qualified DNA samples by PCR. Then, the damaged ends of the fragments were repaired. Both sides of the DNA fragments were connected with a hairpin adapter to obtain a dumbbell (set of horse ring) structure, which is known as SMRTbell. After annealing, the SMRTbell was fixed at the bottom of the ZWM polymerase and used for sequencing. SMRT sequencing was carried out on PacBio RS II instrument (Pacific Biosciences; Menlo Park, CA, USA) using standard protocols. The polymerase reads with lengths less than 50 bp or mass values less than 0.75 were filtered out. Then, the clean data were mapped to the reference genome (AE003852 and AE003853) using the P_Mapping Module of SMRT Analysis software (v2.3.0). The P_Modification Detection (score ≥ 20, coverage≥25 and identificationQv≥20) and P_MotifFind (score ≥ 20 and coverage≥25) Modules were used to identify the methylation sites and motifs. All kinetic raw data from SMRT sequencing have been deposited in NCBI (SRA, PRJNA477395).

### Construction of the gene deletion mutants

To construct the *dcm1*, *dcm2* and *dcm3* gene deletion mutants of strain VC211, two-step overlap PCR was used to generate the fused homologous arms of the target genes. For example, the flanking regions upstream and downstream of *dcm1* were amplified from the genomic DNA of VC211 with two primer sets: del-dcm1–1-*SacI* and del-dcm1–2, and del-dcm1–3 and del-dcm1–4-*SpeI*. After gel purification, the PCR products were mixed, diluted 1000-fold and used as templates for the second round of PCR with the 1 and 4 primers of each gene (primers used in the study are listed in Additional file 3: Table S1). Then, the overlap PCR products and suicide plasmid pWM91were double digested with *SacI* and *SpeI* and were ligated and transformed into SM10. By conjugation and negative selection with 10% sucrose, the *dcm1*, *dcm2* and *dcm3* deletion mutants of VC211 were constructed. Ninety-eight clones on the sucrose plates of each deletion were selected and tested by PCR with the del *dcm*1 and 4 primers of each gene; these clones were further verified by the *dcm*-test-U/L primer sets. The presence of the IncA/C plasmid in the strains was also confirmed by the primer set *repA*-F/R for the plasmid replicase [7], and the primer set *ctxAB*-U/L for the toxic gene *ctxAB* of *V. cholerae* was used as a positive control. On the basis of single-gene deletion, the two-gene deletion mutants VC211∆(*dcm1*,*dcm2*), VC211∆(*dcm2*,*dcm3*), and VC211∆(*dcm1*,*dcm3*) and the three-gene deletion mutant VC211∆(*dcm1*,*dcm2*,*dcm3*) were constructed by the same method.

## Additional files

Additional file 1: **Table S2.** Genes with obviously changed methylation type in SMRT sequencing. (PDF 77 kb)

Additional file 2: **Figure S1.** The methylation percentage of the 9 bases flanking the methylation site of (mC at the fourth base). (PDF 89 kb)

Additional file 3: **Table S1.** Primers used in this study. (PDF 8 kb)

## Data Availability

All the data supporting the findings are presented in the manuscript and the associated supplementary information file. The raw data were deposited into NCBI (SRA, PRJNA477395).

## Abbreviations

DMR: Differentially methylated region
LB: Luria–Bertani
RM: Restriction modification
SMRT: Single-molecule, real-time
