# Supplementary material for: A mobile restriction modification system consisting of methylases on the IncA/C plasmid
Source: Mob DNA. 2019 Jun 7;10:26. doi: 10.1186/s13100-019-0168-1 (PMC6555945; doi:10.1186/s13100-019-0168-1)
Supplement: Supplementary file 1 — Table S2. Genes with obviously changed methylation type in SMRT sequencing. (PDF 77 kb) [file 13100_2019_168_MOESM1_ESM.pdf]

Supplementary table 1.Genes with obviously changed methylation type in SMRT sequencing.

Number	Local tag	C6706		CV2		TIGR Common Name	Gene Symbol	Mainrole	Subrole
		m4C	m6A	m4C	m6A				
1	VC_0025	+	+	+		hypothetical protein			
2	VC_0141	+	+	+		hypothetical protein			
3	VC_0367	+	+	+		primosomal replication protein N	*priB*	DNA metabolism	DNA replication, recombination, and repair
4	VC_0368	+	+	+		ribosomal protein S18	*rpsR*	Protein synthesis	Ribosomal proteins: synthesis and modification
5	VC_0686	+	+	+		hypothetical protein			
6	VC_0912	+	+	+		hypothetical protein			
7	VC_1007	+	+	+		hypothetical protein			
8	VC_1020	+	+	+		hypothetical protein			
9	VC_1109	+	+	+		hypothetical protein			
10	VC_1142	+	+	+		cold shock-like protein CspD	*cspD*	Regulatory functions	Other
11	VC_1227	+	+	+		hypothetical protein			
12	VC_1247	+	+	+		hypothetical protein			
13	VC_1415	+	+	+		hcp protein	*hcp-1*	Unknown function	General
14	VC_1441	+	+	+		cytochrome c oxidase, subunit CcoO	*ccoO*	Energy metabolism	Electron transport
15	VC_1474	+	+	+		conserved hypothetical protein		Hypothetical proteins	Conserved
16	VC_1487	+	+	+		conserved hypothetical protein		Hypothetical proteins	Conserved
17	VC_1569	+	+	+		hypothetical protein			
18	VC_1835	+	+	+		peptidoglycan-associated lipoprotein	*pal*	Cell envelope	Other
19	VC_1962	+	+	+		lipoprotein		Cell envelope	Other
20	VC_2170	+	+	+		hypothetical protein			
21	VC_2200	+	+	+		flagellar basal-body rod protein FlgB	*flgB*	Cellular processes	Chemotaxis and motility
22	VC_2423	+	+	+		fimbrial protein	*pilA*	Cell envelope	Surface structures
23	VC_2575	+	+	+		ribosomal protein L36	*rpmJ*	Protein synthesis	Ribosomal proteins: synthesis and modification
24	VC_2652	+	+	+		conserved hypothetical protein		Hypothetical proteins	Conserved
25	VC_A0019	+	+	+		hypothetical protein			
26	VC_A0028	+	+	+		hypothetical protein			
27	VC_A0045	+	+	+		protease-related protein		Protein fate	Degradation of proteins, peptides, and glycopeptides
28	VC_A0100	+	+	+		phage shock protein E	*pspE*	Cellular processes	Adaptations to atypical conditions
29	VC_A0138	+	+	+		hypothetical protein			
30	VC_A0159	+	+	+		conserved hypothetical protein		Hypothetical proteins	Conserved
31	VC_A0182	+	+	+		sigma-54 dependent transcriptional regulator		Regulatory functions	Other
32	VC_A0313	+	+	+		MutT/nudix family protein		DNA metabolism	DNA replication, recombination, and repair
33	VC_A0318	+	+	+		conserved hypothetical protein		Hypothetical proteins	Conserved
34	VC_A0448	+	+	+		hypothetical protein			
35	VC_A0480	+	+	+		hypothetical protein			
36	VC_A0705	+	+	+		phosphoglycerate transport regulatory protein PgtB, putative		Regulatory functions	Other
37	VC_A0729	+	+	+		hypothetical protein			
38	VC_A0735	+	+	+		hypothetical protein			
39	VC_A0844	+	+	+		hypothetical protein			
40	VC_A0857	+	+	+		hypothetical protein			
41	VC_A0859	+	+	+		oxidoreductase, aldo/keto reductase 2 family		Central intermediary metabolism	Other
42	VC_A0889	+	+	+		transcriptional regulator, LysR family		Regulatory functions	Other
43	VC_A1019	+	+	+		conserved hypothetical protein		Hypothetical proteins	Conserved
44	VC_A1080	+	+	+		secretion protein, HlyD family		Protein fate	Protein and peptide secretion and trafficking
45	VC_t021	+	+	+					
46	VC_t042	+	+	+					
47	VC_t058	+	+	+					

Number	Local tag	C6706		CV2		TIGR Common Name	Gene Symbol	Mainrole	Subrole
		m4C	m6A	m4C	m6A				
48	VC_t085	+	+	+					
49	VC_0001	+	+		+	hypothetical protein			
50	VC_1243	+	+		+	hypothetical protein			
51	VC_1379	+	+		+	hypothetical protein			
52	VC_2304	+	+		+	hypothetical protein			
53	VC_A0253	+	+		+	antibiotic acetyltransferase		Cellular processes	Toxin production and resistance
54	VC_A0463	+	+		+	biphenyl-2,3-diol 1,2-dioxygenase III-related protein		Central intermediary metabolism	Other
55	VC_A0541	+	+		+	hypothetical protein			
56	VC_A1091	+	+		+	chemotaxis protein methyltransferase CheR	*cheR-3*	Cellular processes	Chemotaxis and motility
57	VC_t045	+	+		+				
58	VC_1030	+				hypothetical protein			
59	VC_1387	+				hypothetical protein			
60	VC_1648	+				hypothetical protein			
61	VC_2034	+				hypothetical protein			
62	VC_2230	+				phosphoheptose isomerase	*lpcA*	Cell envelope	Biosynthesis and degradation of surface polysaccharides and lipopolysaccharides
63	VC_2328	+				hypothetical protein			
64	VC_2648	+				hypothetical protein			
65	VC_2752	+				hypothetical protein			
66	VC_A0040	+				conserved hypothetical protein		Hypothetical proteins	Conserved
67	VC_A0109	+				hypothetical protein			
68	VC_A0189	+				response regulator		Regulatory functions	Other
69	VC_A0228	+				vibriobactin and enterobactin ABC transporter, permease protein	*vctD*	Transport and binding proteins	Cations
70	VC_A0301	+				oxidoreductase, short-chain dehydrogenase/reductase family		Central intermediary metabolism	Other
71	VC_A0326	+				hypothetical protein			
72	VC_A0342	+				hypothetical protein			
73	VC_A0578	+				conserved hypothetical protein		Hypothetical proteins	Conserved
74	VC_A0625	+				heme receptor HasR	*hasR*	Transport and binding proteins	Cations
75	VC_A0653	+				PTS system, sucrose-specific IIBC component	*scrA*	Transport and binding proteins	Carbohydrates, organic alcohols, and acids
76	VC_A0672	+				hypothetical protein			
77	VC_A0752	+				thioredoxin 2	*trxC*	Energy metabolism	Electron transport
78	VC_A0888	+				transcriptional regulator, LuxR family		Regulatory functions	Other
79	VC_A0934	+				hypothetical protein			
80	VC_A1047	+				mannitol operon repressor	*mtlR*	Regulatory functions	Other
81	VC_A1066		+			hypothetical protein			
82	VC_0107	+		+	+	hypothetical protein			
83	VC_0226	+		+	+	hypothetical protein			
84	VC_0479	+		+	+	hypothetical protein			
85	VC_0621	+		+	+	hypothetical protein			
86	VC_0805	+		+	+	hypothetical protein			
87	VC_0996	+		+	+	hypothetical protein			
88	VC_1027	+		+	+	molybdenum cofactor biosynthesis protein D	*moaD*	Biosynthesis of cofactors, prosthetic groups, and carriers	Molybdopterin
89	VC_1239	+		+	+	cobinamide kinase/cobinamide phosphate guanylyltransferase	*cobU*	Biosynthesis of cofactors, prosthetic groups, and carriers	Heme, porphyrin, and cobalamin
90	VC_1462	+		+	+	RstB2 protein	*rstB2*	Other categories	Prophage functions
91	VC_1468	+		+	+	conserved hypothetical protein		Hypothetical proteins	Conserved
92	VC_1498	+		+	+	conserved hypothetical protein		Hypothetical proteins	Conserved

Number	Local tag	C6706		CV2		TIGR Common Name	Gene Symbol	Mainrole	Subrole
		m4C	m6A	m4C	m6A				
93	VC_1780	+		+	+	hypothetical protein			
94	VC_1913	+		+	+	conserved hypothetical protein		Hypothetical proteins	Conserved
95	VC_2663	+		+	+	hypothetical protein			
96	VC_A0007	+		+	+	3-hydroxyisobutyrate dehydrogenase, putative		Energy metabolism	Other
97	VC_A0292	+		+	+	hypothetical protein			
98	VC_A0419	+		+	+	hypothetical protein			
99	VC_A0456	+		+	+	hypothetical protein			
100	VC_A0483	+		+	+	hypothetical protein			
101	VC_A0540	+		+	+	formate transporter 1, putative		Transport and binding proteins	Carbohydrates, organic alcohols, and acids
102	VC_A0631	+		+	+	hypothetical protein			
103	VC_A0638	+		+	+	transporter, AcrB/D/F family		Transport and binding proteins	Cations
104	VC_A0658	+		+	+	methyl-accepting chemotaxis protein		Cellular processes	Chemotaxis and motility
105	VC_A0760	+		+	+	arginine ABC transporter, ATP-binding protein	*artP*	Transport and binding proteins	Amino acids, peptides and amines
106	VC_A0803	+		+	+	serine protease, putative		Protein fate	Degradation of proteins, peptides, and glycopeptides
107	VC_A1021	+		+	+	transcriptional regulator, LysR family		Regulatory functions	Other
108	VC_r003	+		+	+				
109	VC_r019	+		+	+				
110	VC_t046	+		+	+				
111	VC_t050	+		+	+				
112	VC_A0244		+	+	+	sugar isomerase SgaE, AraD/FucA family		Energy metabolism	Sugars
113	VC_A0399		+	+	+	hypothetical protein			
114	VC_A0635		+	+	+	transcriptional regulator, LysR family		Regulatory functions	Other
115	VC_A1015		+	+	+	Na+/H+ antiporter	*nhaD*	Transport and binding proteins	Cations
116	VC_A0698	+			+	hypothetical protein			
