# Supplementary material for: A mobile restriction modification system consisting of methylases on the IncA/C plasmid
Source: Mob DNA. 2019 Jun 7;10:26. doi: 10.1186/s13100-019-0168-1 (PMC6555945; doi:10.1186/s13100-019-0168-1)
Supplement: Supplementary file 2 — Figure S1. The methylation percentage of the 9 bases flanking the methylation site of (mC at the fourth base). (PDF 89 kb) [file 13100_2019_168_MOESM2_ESM.pdf]

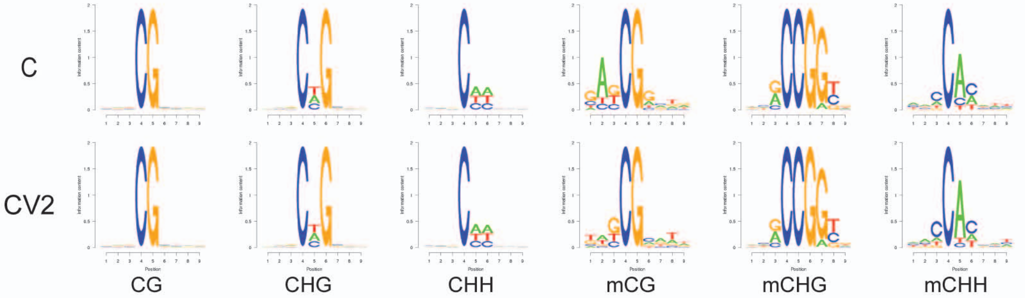

Supplementary figure 1. The methylation percentage of the 9 bases flanking the methylation site of (mC at the fourth base).
