# Supplementary material for: A mobile restriction modification system consisting of methylases on the IncA/C plasmid
Source: Mob DNA. 2019 Jun 7;10:26. doi: 10.1186/s13100-019-0168-1 (PMC6555945; doi:10.1186/s13100-019-0168-1)
Supplement: Supplementary file 3 — Table S1. Primers used in this study. (PDF 8 kb) [file 13100_2019_168_MOESM3_ESM.pdf]

Supplementary table 3. Primers used in this study

Primers	Sequences
kan-844-stuI-U	AAAAGGCCTTGTAGGCTGGAGCTGCTTCG
kan-844-stuI-L	AAAAGGCCTATGGGAATTAGCCATGGTCC
del dcm1-1-SacI	CGAGCTCATAAGCTGTACAGCACTGGTTC
del dcm1-2	CCGTCCCAATCGGGAAGTAATG
del dcm1-3	CATTACTTCCCGATTGGGACGGGAGACGCAATCATGGTCACTTC
del dcm1-4-SpeI	GGACTAGTAAGAGGTACGGAGCTACGCCAG
del dcm2-1-SacI	CGAGCTCAACCGCCTGATCGCTATGGAAG
del dcm2-2	CGATCGGTAACATTGGTCAACC
del dcm2-3	GGTTGACCAATGTTACCGATCGTTGACTGGCGGGGTAGACATTC
del dcm2-4-SpeI	GGACTAGTCTCAGGTTTCGGTGGAACGAAC
del dcm3-1-SacI	CGAGCTCTACTGGAGCAAGCGTAATCC
del dcm3-2	TGAACATGGCGTCCTCCATC
del dcm3-3	GATGGAGGACGCCATGTTCATTCGCATCGAGGTAGGTGTC
del dcm3-4-SpeI	GGACTAGTCCCAGTTCACTGAGCACTAG
ctxAB-L	CTCAGACGGGATTTGTTAGGCACG
ctxAB-U	AATTGCGGCAATCGCATGAG
repA-F	CGTTGGGGTTCATCAATG
repA-R	TACGACGCGAACCAGATC
dcm1-test-U	TGGCGTAACAGCTTGAGCAATCTAC
dcm1-test-L	GGTACTTCTGCATTTTGCATAGTCC
dcm2-test-L	CCCTTGTCCAGAAAACTCAATAACG
dcm3-test-L	GATCTCATCCACCAGCTTCC
kan-mini-U	GTGGAGAGGCTATTCGGCTATG
kan-mini-L	TACTTTCTCGGCAGGAGCAAGG
